# Application of Gross Tissue Response System in Gastric Cancer After Neoadjuvant Chemotherapy: A Primary Report of a Prospective Cohort Study

**DOI:** 10.3389/fonc.2021.585006

**Published:** 2021-11-24

**Authors:** Hua Yang, Wei-Han Zhang, Rui Ge, Bo-Qiang Peng, Xin-Zu Chen, Kun Yang, Kai Liu, Xiao-Long Chen, Du He, Jian-Ping Liu, Wei-Wei Zhang, Yun Qin, Zong-Guang Zhou, Jian-Kun Hu

**Affiliations:** ^1^ Department of Gastrointestinal Surgery and Laboratory of Gastric Cancer, State Key Laboratory of Biotherapy, West China Hospital, Sichuan University, and Collaborative Innovation Center for Biotherapy, Chengdu, China; ^2^ Department of Pathology, West China Hospital, Sichuan University, Chengdu, China; ^3^ Department of Radiology, West China Hospital, Sichuan University, Chengdu, China; ^4^ Department of Gastrointestinal Surgery and Laboratory of Digestive Surgery, State Key Laboratory of Biotherapy, West China Hospital, Sichuan University, and Collaborative Innovation Center for Biotherapy, Chengdu, China

**Keywords:** gastric cancer, advanced, neoadjuvant chemotherapy, gross tissue response, complications, operation time, intraoperative blood loss

## Abstract

**Objective:**

We previously established a gross tissue response (GTR) system to evaluate the intraoperative response of perigastric tissue in patients with gastric cancers to neoadjuvant chemotherapy. This prospective cohort study aims to confirm the relationship between gross tissue response and clinicopathological characteristics and explore the possibility of using the GTR system to predict the difficulty of surgery and the occurrence of postoperative complications within 30 days.

**Methods:**

A total of 102 patients with gastric cancer from January 2019 to April 2020 were enrolled in this study. The degrees of fibrosis, edema, and effusion in the perigastric tissues were assessed intraoperatively according to the GTR system. We systematically analyzed the relations between GTR and clinicopathological characteristics, and then a prediction model that includes GTR was established to predict the difficulty of surgery and the occurrence of postoperative complications within 30 days.

**Results:**

Finally, the study included 71 male patients and 31 female patients. The patients had an average age of 58.79 ± 1.03 years, BMI of 22.89 ± 0.29, and tumor diameter of 4.50 ± 0.27 cm. Among these patients, 17 underwent laparoscopic gastrectomy, 85 underwent open gastrectomy, the average operation time was 294.63 ± 4.84 minutes, and the mean volume of intraoperative blood loss was 94.65 ± 5.30 ml. The overall 30-day postoperative complication rate was 19.6% (20/102). The total GTR was significantly related to the primary tumor stage, operation time and 30-day postoperative complication rate (p<0.05). Edema and effusion were significantly related to intraoperative blood loss (p<0.05). The logistic regression analysis identified that the total GTR score (score: 4-9, OR 2.888, 95% CI: 1.035-8.062, p = 0.043) was an independent risk factor for postoperative complications within 30 days, and the total GTR score (score 4-9, OR 3.32, 95% CI 1.219-9.045, p=0.019) was also an independent risk factor for operation time. The AUC of the total GTR score for predicting postoperative complications within 30 days was 0.681.

**Conclusion:**

According to the results of the present study, the gross tissue response (GTR) system is an effective tool that may be used to predict the risk of a difficult operation after neoadjuvant chemotherapy and postoperative complications. Although neoadjuvant chemotherapy improves the therapeutic effect, it also increases the risk of surgical trauma and postoperative complications.

**Clinical Trial Registration:**

ClinicalTrials.gov, identifier NCT03791268.

## Introduction

Gastric cancer is ranked as the third most common cause of cancer-related death worldwide of digestive system, especially in China (1–3). Neoadjuvant chemotherapy can improve the overall survival rate and disease-free survival rate of locally advanced carcinoma of the esophagus and gastric junction (4). Neoadjuvant chemotherapy can control tumor cell micro metastasis and reduce the risks of tumor recurrence and metastasis, thus leading to survival benefits for patients with locally advanced gastric cancers. The National Comprehensive Cancer Network (NCCN) guidelines for treating gastric cancer recommend neoadjuvant chemotherapy (evidentiary Category 1) as the preferred treatment option for locally advanced gastric cancers (cT2-4Nx) (5, 6). In general, reducing the tumor stage, attaining a higher potential of radical resection and improving overall prognosis are the advantages of neoadjuvant chemotherapy treatment strategies for advanced-stage gastric cancer patients.

The tissue response around the target organ after chemotherapy may increase the difficulty of operations. Regarding gastric cancer surgery, D2 lymphadenectomy is a demanding technique for advanced gastric cancers. Edema, effusion and fibrosis in the perigastric tissue, metastatic lymph nodes, and primary tumor may significantly increase the difficulty of the operation during tissue dissociation and lymph node dissection for gastric cancer. However, whether a correlation exists between the tissue response to chemotherapy and surgical difficulty or postoperative complications after gastrectomy is unclear. Our study group previously established a standard called the gross tissue response (GTR) system to evaluate the degree of fibrosis and edema in the surgical field and intraoperative effusion after neoadjuvant chemotherapy for gastric cancer surgery (7).

Therefore, we conducted this prospective cohort study to explore the relationship between the gross tissue response (GTR) according to our system and postoperative complications for locally advanced gastric cancer patients receiving neoadjuvant chemotherapy.

## Methods

### Study Design

This study was a prospective, observational cohort study. This study was approved by the Ethics Committee of West China Hospital, Sichuan University (2018(No.34)) and registered on ClinicalTrials.gov (ClinicalTrials.gov Identifier: NCT03791268). The present study was reported in line with the STROCSS criteria (8).

### Patient Selection

From January 2019 to April 2020, consecutive patients who met the inclusion criteria in the Department of Gastrointestinal Surgery in our hospital were invited to attend the study. The inclusion criteria were as follows: 1) adult patients (age ≥ 18 and ≤75 years); 2) ECOG physical status score ≤ 2 and ASA score ≤ 3; 3) pathologically diagnosed gastric adenocarcinoma; 4) no serious concomitant disease; 5) definite clinical evidence of locally advanced gastric cancer (cT2-4, N0-3, M0) before chemotherapy; 6) agreement to receive systematic neoadjuvant chemotherapy and subsequently undergo gastrectomy; and 7) signed informed consent. The exclusion criteria were as follows: 1) history of gastric perforation; 2) history an upper abdominal operation (except laparoscopic cholecystectomy); 3) emergency operation due to obstruction, perforation, or acute hemorrhage; 4) inability to endure surgical treatment caused by other serious concomitant diseases; 5) severe mental illness; and 6) request to withdraw from the clinical study after signing the consent form.

### Perioperative Chemotherapy and Evaluation

In this study, diagnostic laparoscopic exploration to clarify the clinical stage and identify occult peritoneal metastasis was not required but recommended for patients before neoadjuvant chemotherapy. The laparoscopic exploration process followed the “four-step method” described in our previous report (9). The neoadjuvant chemotherapy strategy was discussed by the multi-disciplinary team of the Gastric Cancer in West China Hospital. There were no requirements for the neoadjuvant treatment regimens, and generally, at least three cycles of the XELOX regimen (capecitabine was provided at 1000 mg/m^2^, twice a day on days 1–14 and oxaliplatin was provided at 130 mg/m^2^ on day 1) were recommended for patients included in the present study. The chemotherapy toxicity response was evaluated and recorded according to the Common Terminology Criteria for Adverse Events (CTCAE V4.0) (10). For patients who had serious chemotherapy-related adverse events, neoadjuvant chemotherapy was terminated, and they were prepared for surgery. Postoperative chemotherapy was scheduled according to the postoperative pathological evaluation.

Before and after the scheduled neoadjuvant chemotherapy, the patients underwent enhanced abdominal computed tomography (CT) scans to evaluate the clinical stage of the tumors and the effect of neoadjuvant chemotherapy. Clinical tumor regression was measured by two experienced radiologists according to the Response Evaluation Criteria in Solid Tumors (RECIST 1.1) guidelines (11). In addition, it needs to be mentioned that the RECIST 1.1 guidelines were designed for solid tumors, but we modified it to also include the largest regional lymph node as a target lesion, making the guidelines suitable for gastric cancers. The methods for assessing clinical tumor regression with the RECIST guidelines are presented in **Supplementary Data 1**.

### Surgical Treatment and Intraoperative Evaluation

Radical gastrectomy with D2 or D2 plus lymphadenectomy was performed following the Japanese gastric cancer treatment guidelines 2014 (ver. 4) (12). There were no limitations for total gastrectomy or distal gastrectomy in this study. The resection type was determined by the tumor location, tumor margins and status of perigastric lymph nodes according to the Japanese treatment guidelines (12). Exploration of the peritoneal cavity before surgical resection was recommended for patients clinically evaluated as having stable disease (SD) or progressive disease (PD) preoperatively. The indications for laparoscopic gastrectomy were as follows: complete response (CR) or partial response (PR) in the clinical evaluation, a primary tumor size less than 5 cm (before neoadjuvant chemotherapy), and stage ycT2-4a and without bulky regional lymph nodes. Intraoperative frozen sections were routinely analyzed to ensure the safety of the resection margins.

The degree of fibrosis, edema, and effusion in the perigastric tissues was the focus of this study, which was intraoperatively evaluated according to the gross tissue response system from our previous study. Two independent researchers (chief surgeon and first assistant) were responsible for grading the tissue fibrosis, edema, and effusion by general observation. If the score was inconsistent between the two observers, the members of the research group discussed and voted on the final score based on intraoperative photographs or videos. A detailed explanation of the GTR system is presented in **Supplementary Data 2**.

The target areas used to evaluate tissue response were the tissues around the main lymphatic drainage area of the stomach. Specifically, we selected the following target areas from experience based on our previous investigation for the intraoperative evaluation: the greater curvature area (including the greater curvature of the stomach wall and greater omentum); the lesser curvature area (including the lesser curvature of the stomach wall and lesser omentum); the pyloric area (including the tissue and lymph nodes in the supra-pyloric area and infra-pyloric area); the superior area of the pancreas (including the tissue around the left gastric artery, celiac artery, common hepatic artery, and splenic artery.

### Postoperative Evaluation

We collected mesenteric tissue alongside the lesser curvature of the stomach, interstitial tissue alongside the superior margin of the pancreas and interstitial tissue in the infrapyloric area. After pretreatment by a pathologist, all of the collected tissue was made into paraffin sections. Masson’s trichrome staining was carried out to detect the collagen fiber content of these tissue sections. The tumor regression grade after neoadjuvant chemotherapy was evaluated by two experienced pathologists from the pathology department of the West China Hospital according to TRG criteria presented by Becker (12).

### Endpoints and Definitions

The primary endpoint of the present study was the correlation between the severity of the gross tissue response (total score based on the GTR system) and the incidence of postoperative complications within 30 days. The evaluation of gross tissue response was performed according to a previous presentation. The 30-day postoperative complication rate was defined as the incidence of complications during the 30 days after surgery or complications occurring during the same hospitalization, and the occurrence of complications is directly or indirectly related to the operation, not caused by drugs or other treatment measures. The detailed diagnostic criteria for the complications are presented in **Supplementary Data 3**. The severity of postoperative complications was classified according to the Clavien-Dindo classification (10). The secondary aim was to assess whether the GTR system could be used to predict the difficulty of surgery, such as the risk of a prolonged operation time and increased intraoperative bleeding.

### Other Included Clinicopathological Characteristics

The following clinicopathological characteristics were also documented and included in the statistical analysis: age (years), sex (male or female), body mass index (BMI, kg/m^2^), number of chemotherapy cycles, chemotherapy regimen, adverse events due to neoadjuvant chemotherapy, operation type (laparoscopic surgery, open surgery), gastrectomy type (total gastrectomy or partial gastrectomy), operation time (min), intraoperative blood loss (ml), Lauren classification (intestinal type, diffuse type, mix type), Bormann type (types I-IV), tumor location (upper, middle, lower), tumor size (cm), differentiation degree (well, moderated, poor), clinical tumor stage (cTNM stage), number of metastases and harvested lymph nodes, pathological tumor response (according to tumor regression grade, TRG) (13) and pathological tumor stage (ypTNM stage). The pathological examination was performed by two independent pathologists in the Department of Pathology, West China Hospital, according to the 8^th^ TNM staging system for gastric cancer reported by the American Joint Committee on Cancer (14).

### Statistical Analyses

This study hypothesized that the severity of the gross tissue response can be used as an index to predict the incidence of postoperative complications. The area under the ROC curve of the total GTR scores for predicting the 30-day postoperative complication rate was approximately equal to 0.7. The assumed incidence rate of postoperative complications in patients with gastric cancer was 20.7% in our previous study. The estimated sample size was 82, for a power of 90% and two-sided alpha of 0.05, which was calculated with PASS software version 15.0.5 (NCSS LLC, Kaysville, Utah 84037, USA). Finally, we decided to include 102 patients in this study after considering a dropout rate of 20%.

Quantitative variables are expressed as the median and standard deviation (SD). Spearman correlation analysis was used to analyze the relationship among clinicopathological variables. The change in Hounsfield units of the lymph nodes (∆Hu value of lymph nodes) before and after chemotherapy was compared by paired t-tests. The ratio of collagen fiber-stained area to total area was measured to evaluate the content of collagen fibers in the tissues by ImageJ version 1.52a (Wayne Rasband National Institutes of Health, USA). Variables were subjected to univariate analysis and multivariate analysis using logistic regression models with conditional backward step methods to predict the postoperative complications and tumor regression score. The variables tested by univariate analysis that had a P value < 0.20 were included in the multivariate analysis. Receiver operating characteristic (ROC) curves were established to assess the sensitivity and specificity of the predictive values of the total GTR score for postoperative complications and tumor response with the ROC package in R software. In addition, a nomogram was described with the rms package in R software. A two-tailed p value less than 0.05 was regarded as statistically significant. All statistical analyses were performed with R software version 3.5.2 (http://www.r-project.org).

## Results

### Characteristics of the Patients

**Figure 1:**
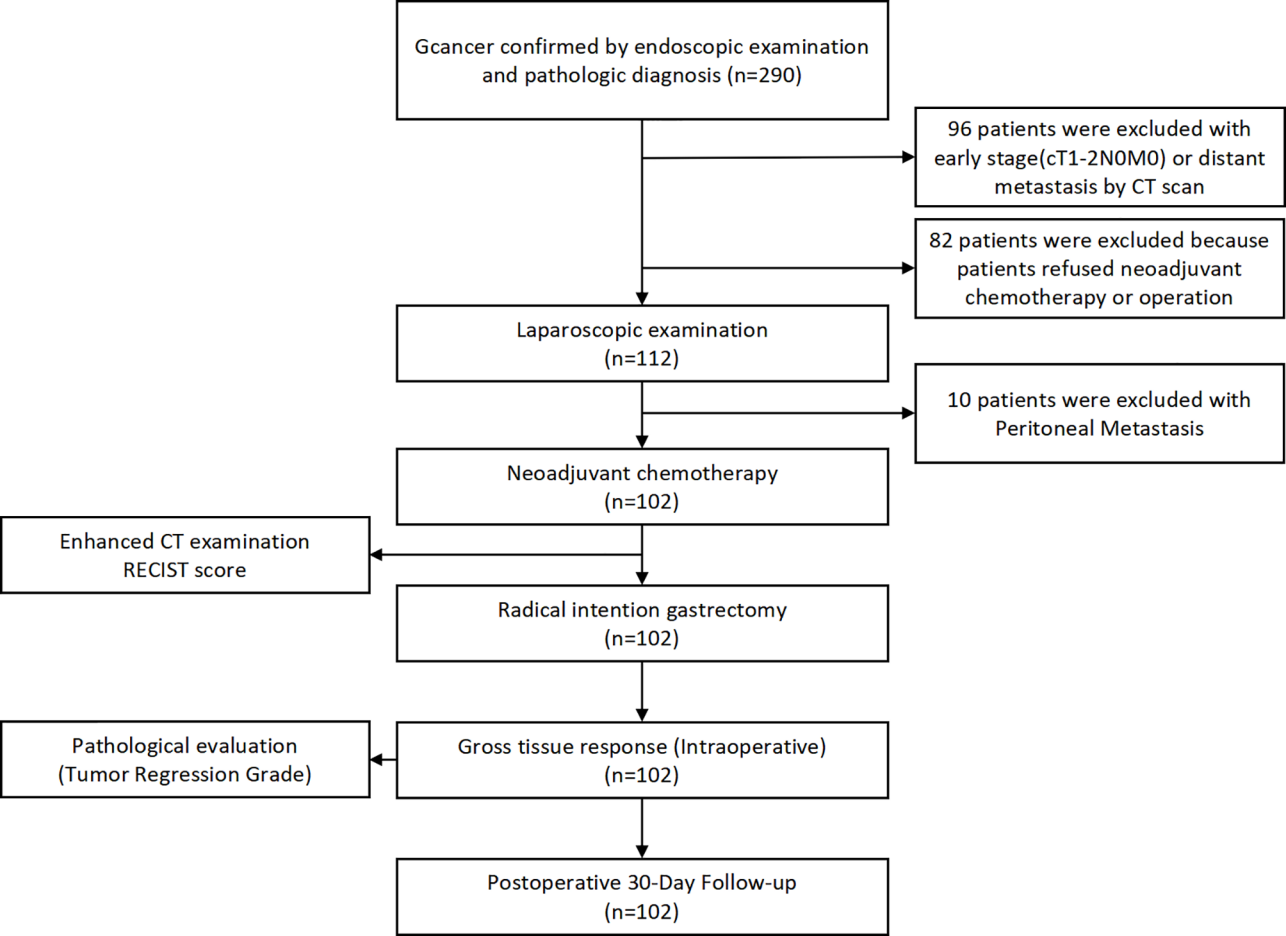
Inclusion and exclusion flow chart for the patients in this study.

A total of 290 primary gastric cancer patients were screened from January 1st, 2019 to April 31st, 2020 in the Department of Gastrointestinal Surgery, West China Hospital, Sichuan University. The screening procedures are presented in **Figure 1**. Finally, 102 gastric cancer patients who received neoadjuvant chemotherapy were included in the present study. The general clinicopathological characteristics of these 102 patients are presented in **Table 1**. In terms of chemotherapy regimen, the majority of patients received the XELOX regimen (88.2%) and received three cycles of treatment (80.4%). Eight patients terminated their scheduled preoperative chemotherapy treatment and turned to surgical treatment due to severe adverse events due to chemotherapy. The average time of postoperative hospital stay was 8.63 ± 6.49 days. There were no patients lost in the postoperative 30-day follow-up. We used Calvien-Dindo Classification grade to reflect the severity of postoperative complication. The overall 30-day postoperative complication rate was 19.6% (20/102). In total, 6.86% (7/102) of patients in grade1, 9.80%(10/102) of patients in grade2, 2.94% (3/102) of patients had greater than grade 3 complications according to the Clavien-Dindo classification. There was no perioperative mortality among the 102 patients. The albumin level reflects the nutritional status of patients, which is 40.57 ± 4.77g/L before chemotherapy and 41.75 ± 3.70g/L after chemotherapy. The results showed that there was no significant difference in albumin level between different grades of edema and effusion (P > 0.05)

**Table 1:** Baseline of clinicopathological variables.

Characteristic		All Patients N=156 (%)	Characteristic		All Patients N = 156 (%)
Age (years)	Mean ± SD	58.79 ± 1.03	Gross tissue response		
Gender	Male	71 (69.6)	Fibrosis grade	0	6 (5.9)
	Female	31 (30.4)		1	48 (47.1)
BMI	Mean ± SD	22.89 ± 0.29		2	41 (40.2)
Tumor location	Cardia	38 (37.3)		3	7 (6.9)
	Body	33 (33.3)	Edema grade	0	9 (8.8)
	Antrum	22 (21.6)		1	59 (57.8)
	Diffuse type	8 (7.8)		2	28 (27.5)
Tumor size	Mean ± SD	4.50 ± 0.27		3	6 (5.9)
Differentiate	Well	0 (0)	Effusion grade	0	7 (6.9)
	Moderate	32 (31.4)		1	62 (60.8)
	Poor	69 (67.6)		2	247 (26.5)
cT stage	cT2	6 (5.9)		3	96 (5.9)
	cT3	37 (36.3)	RECIST score	CR	8 (7.8)
	cT4	59 (57.8)		PR	42 (41.2)
cN stage	cN (-)	9 (8.8)		SD	50 (49.0)
	cN (+)	93 (91.2)		PD	2 (2.0)
cM stage	cM0	93 (91.2)	Tumor regression grade gradedegrscore	0	13 (12.7)
	cM1	9 (8.8)		1	20 (19.6)
TNM stage	2	42 (41.2)		2	54 (52.9)
	3	53 (50.0)		3	15 (14.7)
	4	9 (8.8)	ypT stage	pT0	13 (12.6)
Lauren Classification	Intestinal	43 (42.2)		pT1	17 (16.5)
	Diffuse	23 (22.5)		pT2	16 (15.5)
	Mix	36 (34.3)		pT3	35 (34.0)
Borrmann classification	I	3 (2.9)		pT4	21 (20.4)
	II	21 (20.6)	ypN stage	pN0	48 (46.6)
	III	71 (69.6)		pN1	18 (17.5)
	IV	7 (6.9)		pN2	10 (9.7)
Chemo cycle	< 3	13 (12.7)		pN3	26 (25.2)
	3	72 (80.4)	ypM stage	ypM0	95 (92.2)
	> 3	7 (6.9)		ypM1	7 (6.8)
Chemo regimen	XELOX	90 (88.2)	Number of Positive Lymph nodes	Mean ± SD	4.57 ± 0.81
	Others	12 (11.9)	Number of Examined Lymph nodes	Mean ± SD	41.89 ± 1.33
Operation type	Lap	17 (16.5)	Postoperative Hospital Stay	Days	8.63 ± 6.49
	Open	85 (82.5)	Postoperative 30-day complications	No	82 (80.4)
Resection type	Partial Gastrectomy	43 (42.7)		Yes	20 (19.6)
	Total Gastrectomy	59 (57.3)	Calvien-Dindo Classification**	Grade 1	7 (35.0)
Operation time (min)	Mean ± SD	294.63 ± 4.84		Grade 2	10 (50.0)
Intraoperative blood loss (ml)	Mean ± SD	94.65 ± 5.30		Grade 3	3 (15.0)
Albumin level before NAC (g/L)	Mean ± SD	40.57 ± 4.77		Grade 4	0 (0)
Albumin level after NAC (g/L)	Mean ± SD	41.75 ± 3.70		Grade 5	0 (0)

BMI Body, mass index; RECIST, Response Evaluation Criteria in Solid Tumors; Lap, laparoscopy surgery; Open, open surgery; NAC, neoadjuvant chemotherapy; TRG, tissue regression grade; CR, complete response; PR, partial response; SD, stable disease; PD, progressive disease.**Calvien-Dindo Classification grade reflects the severity of postoperative complication.

### Chemotherapy Response of the Patients

For the gross tissue response assessment, the distribution of fibrosis, edema, and effusion scores are presented in **Table 1**. For the fibrosis scale, the overwhelming majority of patients (grade 1/2, 89, 87.3%) had moderate fibrosis formation. A similar result was also found in the edema and effusion scores. The clinical tumor response assessments adopted the modified RECIST 1.1 classification, and there were 8 (7.8%), 42 (41.2%), 49 (48.0%) and 2 (2.0%) patients evaluated as CR, PR, PD and SD before surgery. Regarding the pathological tumor regression grades among the 102 patients, 13 patients were grade 0, 20 patients were grade 1, 54 patients were grade 2, and 15 patients were grade 3.

### Correlation Between the Total GTR Score and Clinicopathological Characteristics

**Figure 2:**
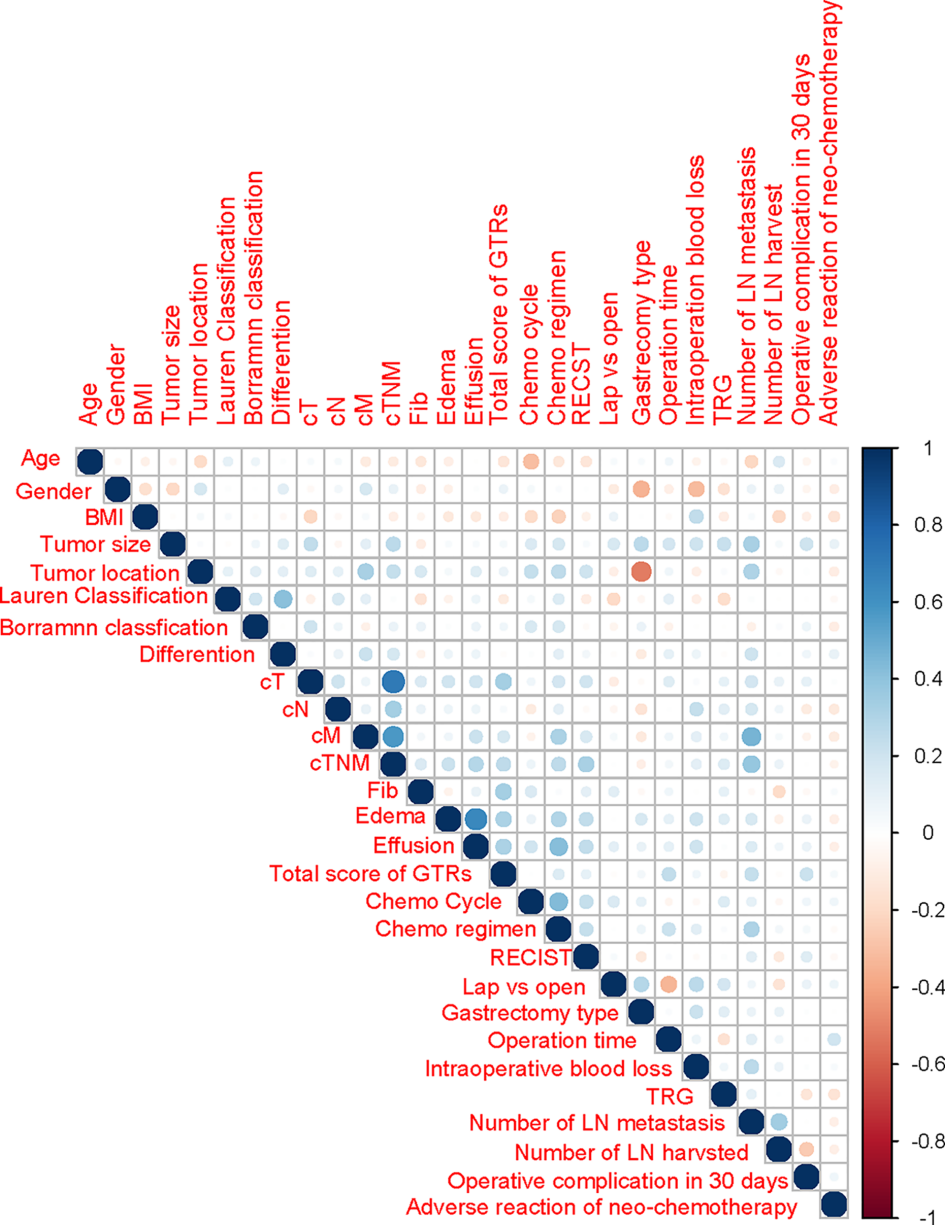
Correlations among independent clinicopathological variables by Spearman analysis. BMI, body mass index; GTR, gross tissue response; Lap, laparoscopy surgery; Open, open surgery; TRS, tissue regression score; RECIST, Response Evaluation Criteria in Solid Tumors; Hu, Hounsfield units; LN, lymph node; NAC, neoadjuvant chemotherapy.

**Figure 3:**
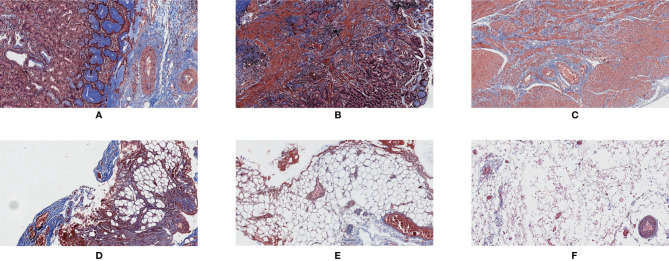
Masson’s trichrome staining. 200×. **(A)** Normal tissue of the gastric wall; **(B)** cancerous tissue; **(C)** tumor tissue showing regression; **(D)** mesenteric tissue alongside the lesser curvature of the stomach; **(E)** interstitial tissue alongside the superior margin of the pancreas; **(F)** interstitial tissue in the infrapyloric area.

Spearman correlation analysis was performed for fibrosis, edema, effusion, total GTR score and other clinicopathological data (**Figure 2** and **Supplementary Table 1**). Edema, intraoperative effusion and total GTR score were significantly related to the cT stage and cTNM stage (p<0.05), and cM stage was correlated with effusion and the total GTR score. In addition, the total GTR score was significantly related to operation time and postoperative complications within 30 days (p<0.05). Edema and effusion were significantly related to intraoperative blood loss (p<0.05). Through pathological tissue sections, we found that the collagen fibers could be dyed blue by Masson’s trichrome staining (**Figure 3**). The average collagen content was significantly correlated with fibrosis, edema and the total GTR score.

### Relationship of GTR With the Difficulty of Surgery and Postoperative Complications

Logistic regression models were set up to evaluate whether clinicopathological variables (including the total GTR score) were risk factors for the incidence of surgical trauma and postoperative complications. The univariate and multivariate analysis results for intraoperative blood loss, operation time and postoperative complications are presented in **Tables 2**–**4**. Finally, multivariate analysis showed that the total GTR score (OR 2.888, 95% CI: 1.035-8.062, p = 0.043) was an independent risk factor for the incidence of postoperative complications. Tumor size (OR 3.104, 95% CI 1.034-9.315, p=0.043), total GTR score (score 4-9, OR 3.32, 95% CI 1.219-9.045, p=0.019), adverse events due to chemotherapy (OR 5.347, 95% CI 1.126-25.655, p=0.035) and operation type (OR 0.066, 95% CI 0.013, p=0.001) were independent risk factors for operation time. The independent risk factors for intraoperative blood loss were BMI and lymph node metastasis, not including GTR score.

**Table 2:** The Univariable and multivariate analysis for the operative complication in 30 days.

Characteristic		Univariable analysis		Multivariable analysis	
		OR (95% CI)	P value	OR (95% CI)	P value
**Age**	<60 *vs*. ≥60	0.742 (0.278-1.981)	0.551		
**Gender (male/female)**	Male *vs*. Female	0.718 (0.236-2.187)	0.560		
**BMI**	<24 *vs*. ≧24	0.636 (0.222- 1.825)	0.400		
**Differentiate**	Well & Moderate *vs*. Poor	1.5( 0.493-4.563)	0.475		
**Lauren classification**	Intestinal				
	Diffuse	1.544 (0.462-5.161)	0.480		
	Mix	0.905 (0.282-2.909)	0.758		
**Borrmann Type**	I				
	II	0.211 (0.013-3.490)	0.277		
	III	0.536 (0.045-6.315)	0.620		
	IV	0.800 (0.044-14.643)	0.880		
**Tumor size**	<4 cm *vs*. ≥4cm	3.289 ( 1.012-10.691)	0.048	3.283 (0.990-10.890)	0.052
**Tumor location**	Cardia				
	Body	1.363 (0.436-4.263)	0.463		
	Antrum	0.699 (0.161-3.035)	0.892		
	Whole	1.476 (0.244-8.915)	0.536		
**Fib**	0-1 *vs* 2-3	0.667 (0.07642-5.873)	0.715		
**Edema**	0-1 *vs* 2-3	2.167 (0.368-12.759)	0.393		
**Effusion**	0-1 *vs* 2-3	2.167 (0.368-12.759)	0.393		
**Total score of GTRs**	0-3				
	4-9	2.893 (1.060-7.898)	0.038	2.888 (1.035-8.062)	0.043
**RECIST**	CR & PR *vs*. PD & SD	2.048 (0.741-5.655)	0.167		
**T stage**	cT2				
	cT3	0.781 (0.075-8.149)	0.781		
	cT4	1.556 (0.167-14.455)	0.698		
**N stage**	cN (-) *vs*. cN (+)	0.447 ( 0.102-1.970)	0.288		
**M stage**	cM0 *vs*. cM1	0.487 ( 0.057-4.134)	0.510		
**TNM stage**	I,II *vs* III,IV	1.383 (0.500-3.827)	0.532		
**Chemo regimen**	XELOX *vs*. Other	1.431 (0.350-5.858)	0.618		
**Chemo cycle**	3 cycles *vs*. Other	1.489 ( 0.468-4.734)	0.500		
**Adverse events of chemo**	No *vs*. Yes	1.632 (0.392-6.806)	0.501		
**Operation type**	Lap *vs* Open	2.015 (0.422-9.632)	0.380		
**Resection type**	Distal gastrectomy				
	Total gastrectomy	0.917 (0.332-2.528)	0.867		
	Proximal gastrectomy	2.000 (0.161-24.916)	0.590		
**Operation time**	<300 min *vs*. ≥300 min	0.953 (0.345-2.630) 1.076(0.377-3.073)	0.992 0.891		
**Intraoperative blood loss**	<100 ml *vs*. ≥100 ml	0.995 (0.373-2.685)	0.992		

OR, Odds ratio; CI, confidence interval;BMI Body, mass index; RECIST, Response Evaluation Criteria in Solid Tumors; Lap, laparoscopy surgery; Open, open surgery; TRG, tissue regression grade; CR, complete response; PR, partial response; SD, stable disease; PD, progressive disease.

**Table 3:** The Univariable and multivariate analysis for the Bloodloss in operation.

Characteristic		Univariable analysis		Multivariable analysis	
		OR (95% CI)	P value	OR (95% CI)	P value
Age	<60 *vs*. ≥60	0.773 (0.350-1.704)	0.523		
Gender (male/female)	Male *vs*. Female	0.247 (0.101-0.602)	0.002	0.343 (0.116-1.016)	0.054
BMI	<24 *vs*. ≧24	2.94 (1.253-7.160)	0.014	3.264 (1.174-9.076)	0.023
Differentiate	Well & Moderate *vs*. Poor	1.459 (0.625-3.407)	0.382		
Lauren classification	Intestinal		0.596		
	Diffuse	0.696 (0.247-1.962)	0.494		
	Mix	0.636 (0.255-1.587)	0.332		
Borrmann Type	I-II *vs*. III-IV	0.818 (0.318-2.096)	0.676		
Tumor size	<4 cm *vs*. ≥4cm	2.374 (1.052-5.357)	0.037	1.886 (0.702-5.062)	0.208
Tumor location	Cardia		0.655		
	Body	0.659 (0.254-1.708)	0.390		
	Antrum	0.520 (0.178-1.518)	0.232		
	Whole	0.867 (0.178-4.210)	0.859		
Fib	0-1 *vs* 2-3	0.929 (0.197-4.383)	0.925		
Edema	0-1 *vs* 2-3	NA	0.999		
Effusion	0-1 *vs* 2-3	NA	0.999		
Total score of GTRs	0-3 *vs* 4-9	1.286 (0.57-2.900)	0.545		
RECIST	CR & PR *vs*. PD & SD	0.909 (0.413-2.001)	0.813		
Operation time	<300 min *vs*. ≥300 min	1.376 (0.620-3.054)	0.433		
Operation type	Lap *vs*. Open	4.400 (1.415-13.678)	0.010	3.373 (0.919-12.379)	0.067
Resection type	Partial *vs*. Total	3.431 (1.500-7.848)	0.003	2.458 (0.893-6.765)	0.082
T stage	cT2		0.736		
	cT3	0.588 (0.096-3.617)	0.567		
	cT4	0.783 (0.132-4.623)	0.787		
N stage	cN (-) *vs*. cN (+)	5.800 (1.140-29.499)	0.034	12.06 (1.896-76.693)	0.008
M stage	cM0 *vs*. cM1	2.642 (0.521-13.404)	0.241		
TNM stage	2		0.362		
	3	1.164 (0.514-2.635)	0.716		
	4	4.957 (0.548-44.844)	0.154		
Chemo regimen	XELOX *vs*. Other	2.294 (0.582-9.042)	0.235		
Chemo cycle	3 cycles *vs*. Other	0.823 (0.307-2.205)	0.699		
Chemo complications	Positive *vs*. negative	1.255 (0.343-4.591)	0.732		


OR, Odds ratio; CI, confidence interval;BMI Body, mass index; RECIST, Response Evaluation Criteria in Solid Tumors; Lap, laparoscopy surgery; Open, open surgery; TRG, tissue regression grade; CR, complete response; PR, partial response; SD, stable disease; PD, progressive disease; NA, not available.

**Table 4:** The Univariable and multivariate analysis for the Operation time.

Characteristic		Univariable analysis		Multivariable analysis	
		OR (95% CI)	P value	OR (95% CI)	P value
Age	<60 *vs*. ≥60	1.279 (0.585-2.795)	0.538		
Gender (male/female)	Male *vs*. Female	0.687 (0.291-1.624)	0.393		
BMI	<24 *vs*. ≧24	0.906 (0.406-2.024)	0.810		
Differentiate	Well & Moderate *vs*. Poor	1.619 (0.687-3.816)	0.271		
Lauren classification	Intestinal		0.298		
	Diffuse	2.194 (0.783-6.148)	0.135		
	Mix	1.594 (0.644-3.946)	0.314		
Borrmann Type	I-II *vs*. III-IV	0.766 (0.276-2.123)	0.608		
Tumor size	<4 cm *vs*. ≥4cm	2.128 (0.939-4.822)	0.070	3.104 (1.034-9.315)	0.043
Tumor location	Cardia		0.111	–	0.3
	Body	2.449 (0.948-6.327)	0.064	2.287 (0.706-7.407)	0.168
	Antrum	0.800 (0.263-2.435)	0.694	0.67 (0.17-2.639)	0.567
	Whole	2.857 (0.591-13.814)	0.192		
Fib	0-1 *vs* 2-3	1.683 (0.357-7.933)	0.511		
Edema	0-1 *vs* 2-3	2.571 (0.449-14.718)	0.289		
Effusion	0-1 *vs* 2-3	2.571 (0.449-14.718)	0.289		
Total score of GTRs	0-3 *vs* 4-9	2.727 (1.202-6.186)	0.016	3.32 (1.219-9.045)	0.019
RECIST	CR & PR *vs*. PD & SD	0.917 (0.420-2.001)	0.827		
Bloodloss in operation	<100 ml *vs*. ≥100 ml	1.376 (0.620-3.054)	0.433		
Operation type	Lap *vs*. Open	0.129 (0.034-0.485)	0.002	0.066 (0.013-0.322)	0.001
Resection type	Partial *vs*. Total	0.655 (0.296-1.445)	0.294		
T stage	cT2		0.351		
	cT3	0810 (0.404-35.905)	0.243		
	cT4	4.833 (0.532-43.921)	0.162		
N stage	cN (-) *vs*. cN (+)	1.029 (0.260-4.709)	0.967		
M stage	cM0 *vs*. cM1	1.585 (0.400-6.283)	0.512		
TNM stage	2		0.701		
	3	1.416 (0.625-3.210)	0.405		
	4	1.103 (0.219-5.567)	0.906		
Chemo regimen	XELOX *vs*. Other	4.297 (1.089-16.953)	0.037	3.078 (0.608-15.577)	0.174
Chemo cycle	3 cycles *vs*. Other	0.772 (0.286-2.086)	0.610		
Chemo complications	Positive *vs*. negative	3.719 (0.926-14.944)	0.064	5.374 (1.126-25.655)	0.035

OR, Odds ratio; CI, confidence interval;BMI Body, mass index; RECIST, Response Evaluation Criteria in Solid Tumors; Lap, laparoscopy surgery; Open, open surgery; TRG, tissue regression grade; CR, complete response; PR, partial response; SD, stable disease; PD, progressive disease.

**Figure 4:**
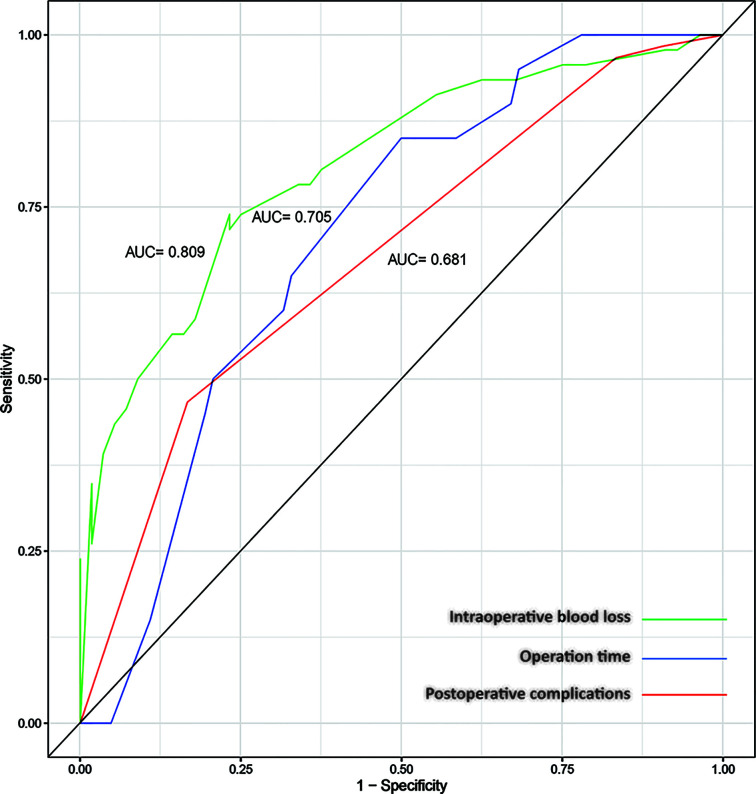
ROC curves for the prediction of intraoperative blood loss (AUC=0.809), operation time (AUC=0.705) and postoperative complications within 30 days (AUC=0.681).

**Figure 5:**
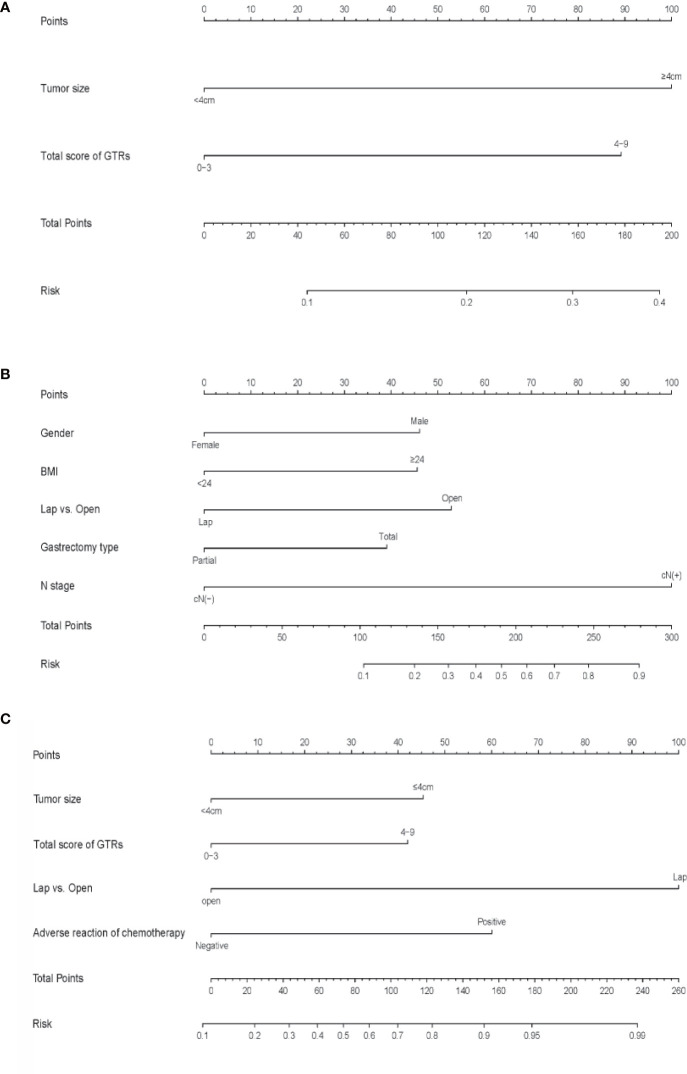
**(A)** Nomogram for predicting postoperative complications within 30 days, **(B)** intraoperative blood loss **(B)** and **(C)** operation time. To calculate the probability, points for each variable are assigned to the corresponding values from the “points” axis, and the sum of points is plotted on the “total points” axis. The probability is the value indicated by a vertical line from the corresponding total points. (BMI, body mass Index; GTRs, gross tissue response system; TRG, tumor regression grade; L, lower; U, upper; M, middle; LN, lymph node.

ROC curves were drawn to evaluate the sensitivity and specificity of the GTR system in the prediction of postoperative complications and difficulty of surgery. According to the results of the logistic regression, ROC curves were constructed, and the AUCs were 0.681, 0.705 and 0.809 for predicting postoperative complications within 30 days, operation time and intraoperative blood loss, respectively (**Figure 4**). To better understand the relationship between the GTR system and the two outcomes, nomograms were established to visualize the logistic regression models of postoperative complications within 30 days (**Figure 5A**), intraoperative blood loss (**Figure 5B**), and operation time (**Figure 5C**). With all the above results, we noticed that high GTR scores were associated with a higher incidence of postoperative complications within 30 days.

## Discussion

For locally advanced gastric cancers, neoadjuvant chemotherapy combined with radical gastrectomy is the preferred treatment, which is recommended by the NCCN guidelines (5). On the one hand, neoadjuvant chemotherapy can achieve tumor down staging, improves the radical resection potential and prolongs the prognosis of advanced-stage gastric cancer patients. On the other hand, the adverse reactions to chemotherapy drugs and tissue response after chemotherapy can increase the difficulty of surgery and risk for postoperative complications. Therefore, we previously established the gross tissue response system, which includes fibrosis, edema and effusion scales, to evaluate the gross tissue response of both the potentially metastatic lymph node area and the surrounding normal tissue area of the stomach after chemotherapy (7). In the present study, we found that the established gross tissue response score was significantly correlated with the primary tumor stage, operation time, intraoperative blood loss and postoperative complications.

The hypothesis of the present study is that we can use this evaluation system to predict the incidence of postoperative complications within 30 days. Generally, the incidence rate of postoperative complications after neoadjuvant chemotherapy is relatively higher than that after surgery alone. Previous prospective studies reported that the postoperative complication rates of patients who received neoadjuvant chemotherapy ranged from 25.7% to 45.7% (15–17). We found a similar postoperative complication rate (19.6%) and rate of severe complications (2.94%) as previous reports. Surgical trauma and myelosuppression after chemotherapy may be reasons that contribute to the high overall incidence rate of postoperative complications within 30 days (18, 19). In the present study, we found that the gross tissue response score was an independent risk factor that could be used to predict the incidence of postoperative complications (AUC = 0.681). This obscures field for tissue dissection and significantly increases the difficulty of lymph node dissection in gastrectomy. Additionally, the edema and effusion response may lead to an increase in the incidence of tissue laceration and capillary bleeding. Moreover, during the tissue dissection process, a large amount of fluid in the tissue can also increase the amount of bleeding during operation. To clear the field of vision, repeated suction and hemostasis processes are needed but will significantly prolong the operation time, thus increasing the trauma of the operation. Therefore, these findings can illustrate why the gross tissue response is related to the difficulty of the operation and postoperative complications.

Tumor regression after neoadjuvant therapy is commonly used to predict the prognosis of cancer patients (20, 21). Becker et al. presented that histological tumor regression after chemotherapy can provide objective and valuable prognostic information (22). Several important clinical studies have shown that patients with pathological complete response (pCR) after neoadjuvant chemotherapy have better overall survival than those with non-pCR (22–25). However, this view remains controversial (26). The present study adopted the tumor regression grade to evaluate pathological tumor regression, which is recommended by the NCCN gastric cancer guidelines (27). A previous study showed that the overgrowth of fibrosis on tumor cells was the major sign of histological tumor regression due to chemotherapy (28). We hypothesized that there was a relationship between gross tissue response and pathological tumor regression. However, while our results showed that gross tissue response was correlated with the cT, cM and cTNM stages, there was no correlation between the GTR score and TRG grade, which means that compared with the degree of tumor regression, the primary burden of the tumor may be more related to tissue response after neoadjuvant chemotherapy. The reason for these phenomena may be that pathological regression grade only evaluates the tumor tissue, and chemotherapy is a systemic treatment that may cause tissue and organ reactions throughout the whole body, which is why we needed to create a brand new system to evaluate gross tissue response.

In the present cohort study, Masson’s trichrome staining was used to detect the content of collagen fibers in the tissues. We found that there was a correlation between the collagen fiber content in the interstitial tissues around the stomach and the fibrosis grade based on the GTR system. This suggested that the criteria of the GTR system could reflect the changes in the interstitial tissues around the stomach after neoadjuvant chemotherapy. In addition, with Masson’s trichrome staining, we observed that the normal tissue in the gastric wall had a clear structure, and collagen fibers were evenly distributed along the gastric wall (**Figure 3A**). However, the opposite was observed in tumor tissue and tumor tissue with regression (**Figures 3B, C**); not only was the structure of the gastric wall disorganized, but the distribution of collagen fibers was also disordered. These phenomena might indicate that neoadjuvant chemotherapy could lead to aseptic inflammation and tumor cell apoptosis, resulting in fibrous tissue hyperplasia in the local microcirculation.

The limitations of this study are as follows: 1) this is a pilot study of the GTR system in patients with gastric cancers. External consistency needs to be explored and validated in further multicenter studies. 2) The present study only analyzed the clinical implications of gross tissue response after neoadjuvant chemotherapy in patients with gastric cancers, and whether GTR system is suitable for patients with other malignant diseases is unclear. 3) The primary endpoints are the relation between the GTR score and short-term postoperative complications in gastric cancer patients receiving neoadjuvant chemotherapy. The lack of long-term follow-up and survival information to explore the relationship between the GTR score and prognosis is another limitation of this study.

## Conclusions

Therefore, according to the results of the present study, the gross tissue response system (GTR) is an effective tool that may be used in the prediction of the difficulty of surgery after neoadjuvant chemotherapy and postoperative complications. Although neoadjuvant chemotherapy improves the therapeutic effect, it also increases the risk of surgical trauma and postoperative complications. Additionally, further studies are needed to explore whether this system is suitable for patients with other malignant diseases receiving neoadjuvant therapy.

## Data Availability Statement

The raw data supporting the conclusions of this article will be made available by the authors, without undue reservation.

## Ethics Statement

The studies involving human participants were reviewed and approved by the Ethics Committee of West China Hospital, Sichuan University (2018(No.34)). The patients/participants provided their written informed consent to participate in this study.

## Author Contributions

W-HZ, Z-GZ, and J-KH designed the study. HY, W-HZ, RG, B-QP, X-ZC, KY, KL, and X-LC collected the clinical information. HY, W-HZ, RG, B-QP, and J-KH conducted the statistical analysis and interpreted the data. DH and J-PL conduct pathological evaluation. W-WZ and YQ conduct radiological evaluation. Z-GZ and J-KH supervised this study. All authors contributed to the article and approved the submitted version.

## Funding

Department of Sichuan Science & Technology Program (20YYJC3357, 2021YJ0475); 135 project for disciplines of excellence, West China Hospital, Sichuan University (ZYJC21006); National Natural Science Foundation of China (81902437); Ten Thousand Talent Program of Sichuan Province (No. TJZ201906).

## Conflict of Interest

The authors declare that the research was conducted in the absence of any commercial or financial relationships that could be construed as a potential conflict of interest.

## Publisher’s Note

All claims expressed in this article are solely those of the authors and do not necessarily represent those of their affiliated organizations, or those of the publisher, the editors and the reviewers. Any product that may be evaluated in this article, or claim that may be made by its manufacturer, is not guaranteed or endorsed by the publisher.

## Abbreviations

GTR, Gross tissue response; NCCN, National Comprehensive Cancer Network; SD, Stable disease; PD, progressive disease; CR, Complete response; PR, Partial response; BMI, Body mass index; TRG, Tumor regression grade; RECIST, Response Evaluation Criteria in Solid Tumors; AUC, Area under curve; OR, Odds ratio; 95% CI, 95% confidential intervals; SD, standard difference.
