# Supplementary material for: Application of Gross Tissue Response System in Gastric Cancer After Neoadjuvant Chemotherapy: A Primary Report of a Prospective Cohort Study
Source: Front Oncol. 2021 Nov 24;11:585006. doi: 10.3389/fonc.2021.585006 (PMC8651877; doi:10.3389/fonc.2021.585006)
Supplement: Supplementary file 1 [file DataSheet_1.docx]

**Supplementary Data 3. Assessment of postoperative 30-day complications**

The present study analyzed postoperative 30-day complications. Postoperative 30-day complications defined as complications incidence during the period of postoperative 30 days or complications happen during the same hospitalization. Postoperative 30-day outpatients were demanded for patients discharge after the operation. The severity of postoperative 30-day complications was evaluated according to the Clavien-Dindo Classification. Following complications were recorded and analyzed in the present study:

1. Incision complications (Dehiscence of abdominal incision, Incisional hernia, Superficial surgical site infection).
2. Abdominal cavity complications (Abdominal or Pelvic cavity infections, Peritoneal effusion or Abscess formation, Intra-abdominal hemorrhage).
3. Anastomotic complications (Anastomotic bleeding or Gastrointestinal active bleeding, Anastomotic leakage, Duodenal stump leakage, Intestinal fistula, Anastomotic stenosis).
4. Obstruction (Afferent loop obstruction, Intestinal obstruction, Internal hernia).
5. Chylous fistula, Bile fistula and Pancreatic fistula.
6. Gastroparesis, intestinal paralysis.
7. Dumping syndrome.
8. Acute pancreatitis, acute cholecystitis.
9. Deep venous thrombosis.
10. Kidney failure, Liver failure, Cardiovascular and Cerebrovascular events (including thrombosis, embolism), Septicopyemia, Systemic Inflammatory Response Syndrome (SIRS), Multiple Organ Dysfunction Syndrome (MODS).
11. Postoperative pulmonary complications (pneumonia, pleural effusion, pulmonary embolism, Acute Respiratory Distress Syndrome (ARDS)).
