# Supplementary figures and images for: Application of Gross Tissue Response System in Gastric Cancer After Neoadjuvant Chemotherapy: A Primary Report of a Prospective Cohort Study
Source: Front Oncol. 2021 Nov 24;11:585006. doi: 10.3389/fonc.2021.585006 (PMC8651877; doi:10.3389/fonc.2021.585006)

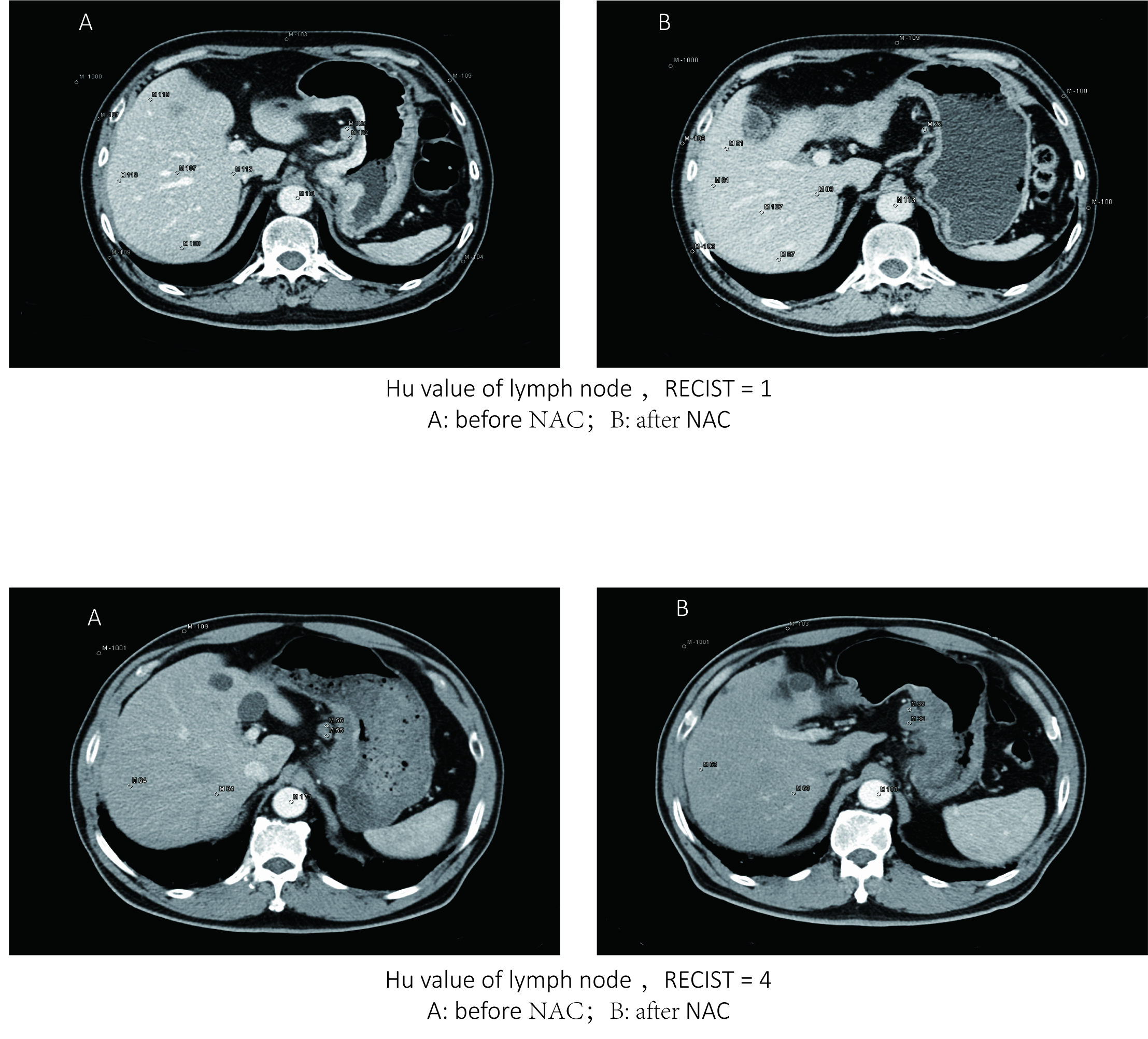

Supplement: Supplementary file 2 [file Image_1.tif]

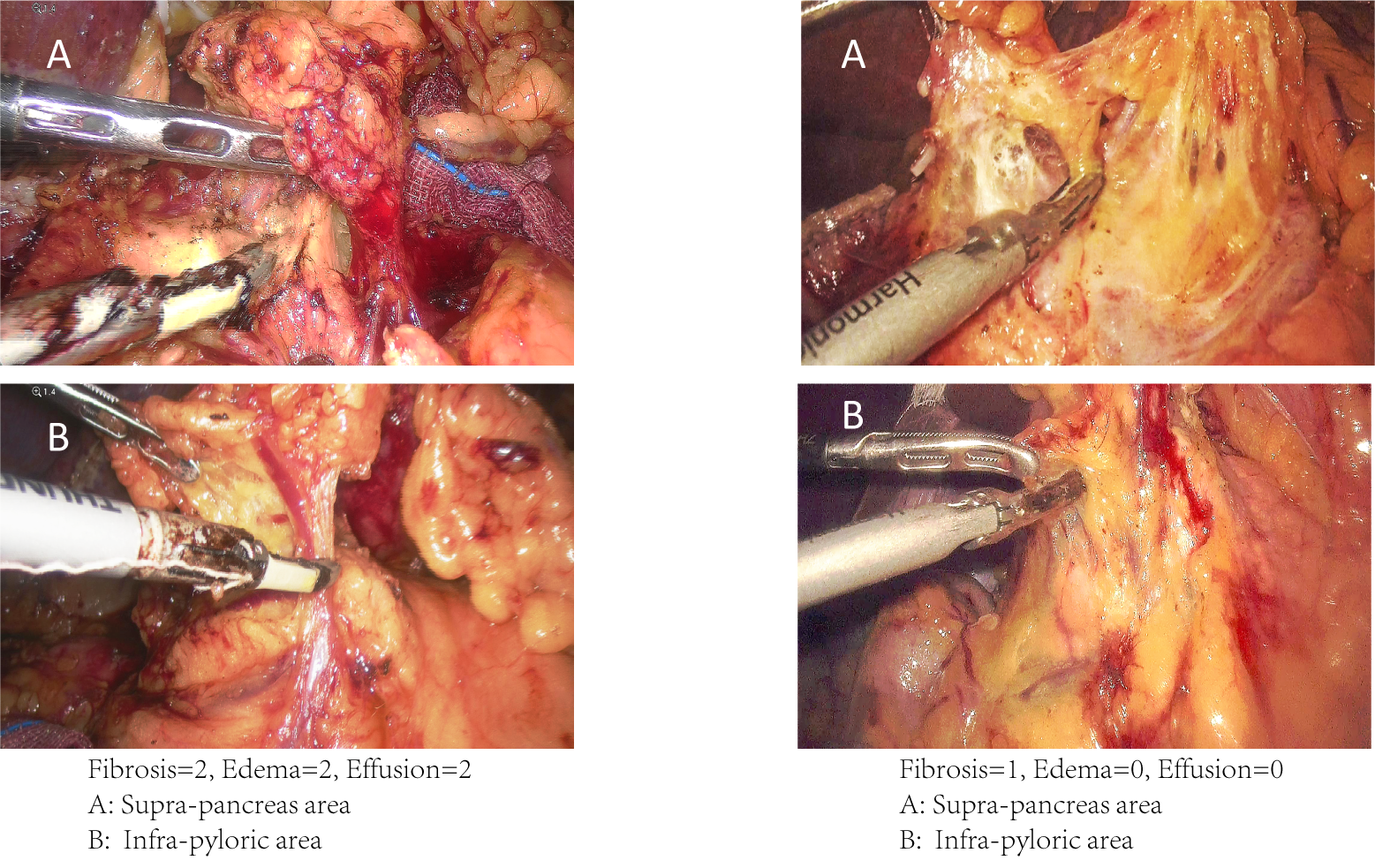

Supplement: Supplementary file 3 [file Image_2.tif]
